# Eudragit^®^: A Versatile Family of Polymers for Hot Melt Extrusion and 3D Printing Processes in Pharmaceutics

**DOI:** 10.3390/pharmaceutics13091424

**Published:** 2021-09-08

**Authors:** Juliana dos Santos, Guilherme Silveira da Silva, Maiara Callegaro Velho, Ruy Carlos Ruver Beck

**Affiliations:** 1 Programa de Pós-Graduação em Ciências Farmacêuticas, Faculdade de Farmácia, Universidade Federal do Rio Grande do Sul, Porto Alegre 90610-900, Brazil; santos.juliana@ufrgs.br (J.d.S.); maiaracvelho@gmail.com (M.C.V.); 2 Departamento de Produção e Controle de Medicamentos, Faculdade de Farmácia, Universidade Federal do Rio Grande do Sul, Porto Alegre 90610-900, Brazil; guilhermemb.500@gmail.com

**Keywords:** additive manufacturing, caplets, drug release, dissolution, polymethacrylate, printlets, tablets

## Abstract

Eudragit^®^ polymers are polymethacrylates highly used in pharmaceutics for the development of modified drug delivery systems. They are widely known due to their versatility with regards to chemical composition, solubility, and swelling properties. Moreover, Eudragit polymers are thermoplastic, and their use has been boosted in some production processes, such as hot melt extrusion (HME) and fused deposition modelling 3D printing, among other 3D printing techniques. Therefore, this review covers the studies using Eudragit polymers in the development of drug delivery systems produced by HME and 3D printing techniques over the last 10 years. Eudragit E has been the most used among them, mostly to formulate immediate release systems or as a taste-masker agent. On the other hand, Eudragit RS and Eudragit L100-55 have mainly been used to produce controlled and delayed release systems, respectively. The use of Eudragit polymers in these processes has frequently been devoted to producing solid dispersions and/or to prepare filaments to be 3D printed in different dosage forms. In this review, we highlight the countless possibilities offered by Eudragit polymers in HME and 3D printing, whether alone or in blends, discussing their prominence in the development of innovative modified drug release systems.

## 1. Introduction

The search for innovative technologies in the production of new medicines is constant and involves techniques that are able to improve the physicochemical and bioavailability characteristics of drugs and increase the patient’s acceptance, among others. In the last two decades, hot melt extrusion (HME) has been extensively used as a technique for manufacturing solid dosage forms [1], especially linked to three-dimensional (3D) printing [2]. More specifically, the fused deposition modelling (FDM) 3D printing technique, where the melt material is deposited layer-by-layer to form an object, has been closely related to the HME process. Thus, besides acting as a technique to produce pharmaceutical products itself, HME has been substantially used as a first step in the production of filaments for FDM [3] or even during the feeding of the materials in some 3D printers.

In the HME process, the mixture of polymers and drugs, as raw material, is passed through a heated barrel with the help of a screw, which can be single or twin, and comes out through a die in a variety of forms, which can be controlled by the operator [3]. In some cases, excipients like plasticizers are necessary to facilitate the material extrusion [4]. The temperature used in HME is usually above the glass transition (T_g_) and melting (Tm) temperature of the chosen polymer. This process favours the mixing of drug and polymer at a molecular level, and its use is justified by a number of advantages, such as being a solvent-free technique, with few steps until the final product, and being easily automated, which is desired by the industry [5].

HME has been applied using a wide range of polymers in the production of different drug delivery systems, such as tablets [6,7], pellets [8,9], implants [10] and transdermal systems [11,12], but has been especially focused on the production of solid dispersions (SDs) of poorly water-soluble drugs [4]. However, in the last few years, its use in pharmaceutics has been boosted even more due to it being linked to 3D printing processes in the development of innovative medicines.

The application of 3D printing techniques in the development of drug delivery systems emerged in the pharmaceutical market after 2015, when the Food and Drug Administration (FDA) approved the first medicine produced with this technology. In a 3D printing process, a material is deposited layer-by-layer to form an object with a unique structure, thus facilitating the customization of doses and therapies [13]. Depending on the type of material and the way that the material is deposited, different 3D printing techniques can be explored. The American Society for Testing and Materials classified the techniques into seven categories (material jetting, material extrusion, vat photopolymerization, powder bed fusion, binder jetting, sheet lamination and directed energy deposition) [14], but not all have been applied in the development of drug delivery systems. The most used techniques for the development of medicines are the extrusion techniques—semisolid extrusion and FDM, stereolithography, inkjet printing and selective laser sintering (SLS) [15]. Thanks to the versatility of 3D printing in the development of personalized medicines, it has been used in the production of the most diverse pharmaceutical forms, such as oral delivery devices (tablets, caplets, and printlets) [16,17,18,19], skin products (films, microneedles and patches) [20,21], implants [22,23], and scaffolds [24,25], among others.

**Figure 1:**
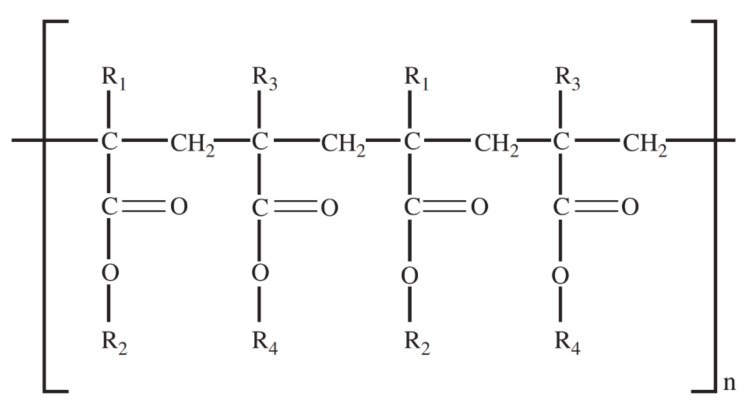
Main structural skeleton of Eudragit polymers.

In both HME and 3D printing, one of the first steps in producing the drug delivery system is to choose a polymer with suitable properties, depending on the desired goal and the designed delivery profile. Polymethacrylates are synthetic polymers of dimethylaminoethyl methacrylates, methacrylic acid and methacrylic acid esters in varying ratios, which are commercially available under different brands [26]. They have been highly used in HME and 3D printing of pharmaceutics. Eudragit^®^ polymers are one of the most famous representatives of polymethacrylates. The Eudragit family has the same common structure (Figure 1), and differ from each other by their substituents, which confer different chemical properties, as can be observed in Table 1. In general, Eudragit polymers are divided into cationic, anionic and neutral and are available as powders, granules, aqueous dispersions and organic solutions [27]. More details about some representatives of this polymer family are presented below.

Eudragit E (EE) is a cationic copolymer and is soluble in gastric pH (up to 5) [27]. This polymer presents fast dissolution at the aforementioned pH because of the hydration of its dimethylamino groups, which are fully protonated at this condition [29]. It is commonly used for the formulation of SDs, sublingual and topical preparations, and tablets with modified characteristics [30,31,32,33].

Eudragit RL (ERL) is a permeable and cationic polymer. Its permeability is provided by the salt ammonium groups in its structure, being more intensely observed as the amount of ammonium groups increases [34]. ERL is composed of methyl methacrylate, ethyl acrylate and a lower percentage (10%) of methacrylic acid ester with quaternary ammonium groups. This polymer is chemically stable and has excellent extrudability. Furthermore, it is insoluble in water and shows pH-independent swelling properties, being highly permeable, as described above [35,36].

On the other hand, Eudragit RS (ERS) has the same molecular structure and the same particularities as ERL, with the exception of its permeability, which is much lower [37]. The only difference between ERS and ERL is, therefore, their ammonium functional group content, which controls the permeability of the polymers [38]. ERS has 5% of quaternary ammonium groups, which is 2× lower than ERL. These two types of polymers (ERL and ERS), with different permeabilities, are often used together in different proportions to achieve the target specific permeability and the desired rate of intestinal absorption. These polymers, alone or in blends, have been used in pharmaceutics for the development of micro [39,40] and nanoparticles [41,42,43,44], coated tablets [45] and mucoadhesive buccal films [46], among others, mainly to obtain sustained release delivery systems.

Eudragit S100 (ES100), Eudragit L100 (EL100) and Eudragit L100-55 (EL100-55) are anionic polymers consisting of poly(methacrylic acid-co-acrylates). The difference between ES100 and EL100 is their active carboxylic group. In ES100, the active carboxylic groups represent 29.2% of its molecular weight, while these groups represent 48.3% in EL100, affording different pH-dependent solubility profiles. On the other hand, EL100-55 is a copolymer composed of methacrylic acid/ethyl acrylate. ES100, EL100 and EL100-55 polymers dissolve above pH 7.0, 6.0 and 5.5, respectively [47,48], and the enteric coating is one of their most recognized applications [28].

Lastly, Eudragit FS 30 D (EFS30D) is an anionic polymer composed of methyl acrylate, methyl methacrylate and methacrylic acid. It is available as a 30% aqueous dispersion, presenting low viscosity and solubility above pH 7.0, and has been used for the formulation of colonic drug delivery systems [26,28].

Although this polymer family has been widely used in the formulation and production of drug delivery systems and pharmaceutical products, as previously reviewed by other groups [26,27], the application of these polymers to the development of innovative devices by 3D printing has recently started in pharmaceutics and has not been reviewed yet. In addition, their use in 3D printing is strongly connected to the HME process. Therefore, this review addresses the main use and applications of the polymers of the Eudragit family in the development of modified drug delivery systems produced by HME and/or 3D printing processes, discussing their versatility and challenges on this topic.

## 2. Current Scenario

In this review, a panoramic view of the use of the Eudragit series in HME and 3D printing is presented. Therefore, scientific articles published between 2011 and June 15th 2021—representing ten years of research—were searched in two databases: Web of Science (keywords: ((3D printing or hot melt extrusion) and drug and eudragit)) and Scopus (keywords: (3D printing and drug and eudragit); (hot melt extrusion and drug and eudragit)). The variations of Eudragit name, such as “methacrylate”, “*methacrylate” and “*methacry*”, as well as the keyword “additive manufacturing” were also used to cover the largest possible number of articles. After a careful analysis of the output of these searches, 122 articles fit our criteria, as referenced below, and were included in this review.

**Figure 2:**
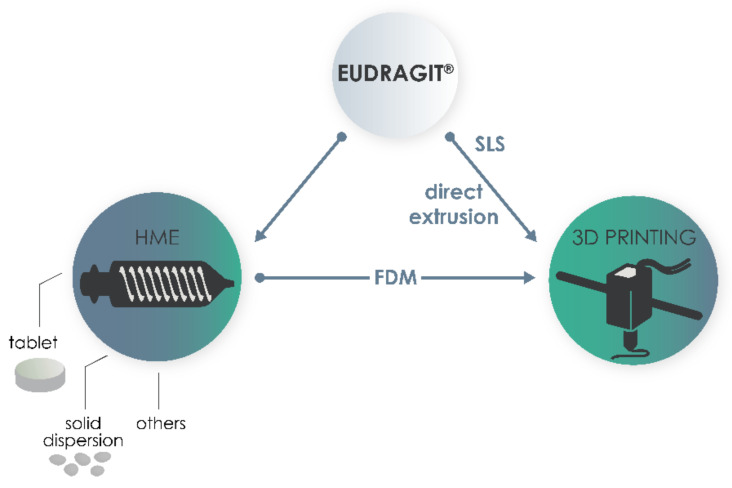
Decision path used to classify articles in the scope of this review. FDM, fused deposition modelling; HME, hot melt extrusion; SLS, selective laser sintering.

Figure 2 represents the decision path for the classification of articles found during these searches. All articles that used Eudragit were included, whether alone or in combination with another representative of the Eudragit family, or even combined with another class of polymer. The selected papers were classified under two big umbrellas: HME and 3D printing. It is important to note that most articles that are based on studies comprising the use of FDM as the 3D printing technique use an extrusion process to produce the filament. In this case, these articles were classified as 3D printing due to their final goal.

**Figure 3:**
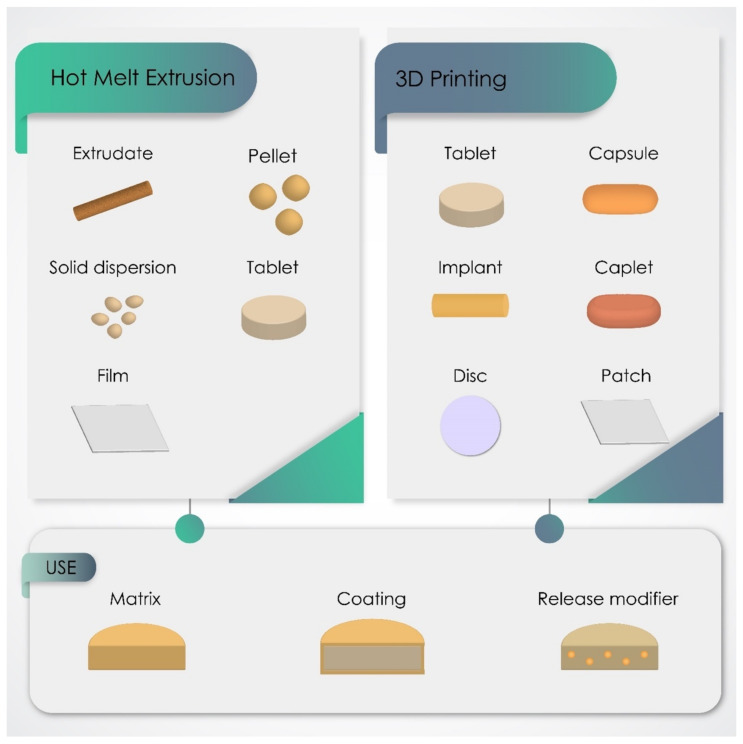
The dosage forms most produced by hot melt extrusion and 3D printing using Eudragit polymers and their main polymeric role in these formulations.

**Figure 4:**
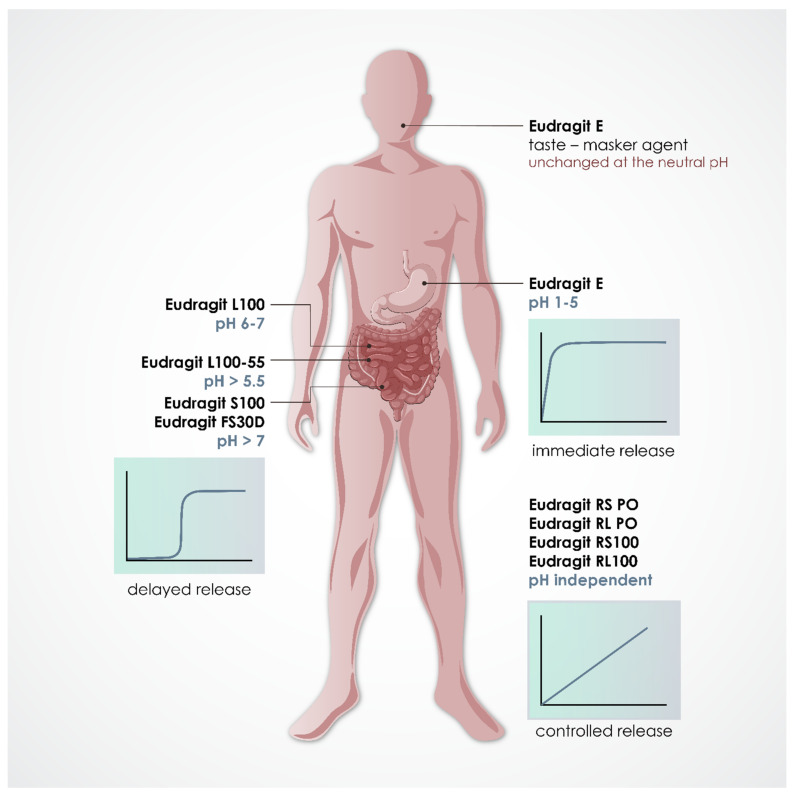
Predictable drug release site from dosage forms produced with Eudragit polymers, according to their physicochemical properties.

The obtained data showed that EE was the most used Eudragit, being used in 50.8% of the 122 studies comprised in this review, followed by ERS (22.9%), ERL (19.7%) and L100-55 (17.2%). The sum of the individual percentages exceeds 100% as some articles used more than one type of Eudragit. Figure 3 presents the different dosage forms produced by HME and 3D printing, with special emphasis on SDs, extrudates, pellets, tablets and caplets. In most of these formulations, Eudragit had the main function of forming the polymeric matrix, in mixtures with other types of Eudragit or with polymers from other families. However, Eudragit was also reported as a coating material and/or release modifier in some studies. The changes in chemical composition of the representative members of the Eudragit family and, consequently, their different physicochemical properties make these polymers suitable for designing customized drug delivery behavior, such as immediate, controlled, sustained, or delayed profiles, as shown in Figure 4. On the other hand, this review does not classify the release profiles reported in the original studies according to these terms because it was difficult to assure a correct classification in some of them, considering the data available and the terms used by their own authors.

## 3. Hot Melt Extrusion

HME has been extensively used in the pharmaceutical industry, mainly to improve the solubility of poorly soluble drugs. In the context of this review, about 82 of the 122 papers, representing (67.2%), were devoted to producing pharmaceutical formulations by HME. Overall, EE has a special prominence in HME, being reported in 54.9% of the 82 articles, mostly to obtain immediate release or taste masked formulations, followed by ERS (20.7%) and EL100-55 (17.1%), which were reported in studies aimed at obtaining sustained and delayed release formulations, respectively.

These 82 articles were classified according to their goal, as follows: to produce SDs (46.3%) (Table 2), to produce solid dosage forms such as tablets in an additional step (17.1%) (Table 3), and to produce other formulations, like pellets (9.7%), films (3.6%) and floating formulations (3.6%) (Table 4). The main scientific findings and contributions of these studies will be discussed in the next sections, following the classification presented above.

### 3.1. Solid Dispersions (SDs)

SDs are characterized by the dispersion of drug molecules in a system, usually a polymeric material [86]. They have been widely used as a strategy to improve the solubility of poorly water-soluble drugs (class II of the biopharmaceutical classification system—BCS), their physical stability, bioavailability and also to cover the bad taste or smell of drugs [63,84,122]. These advantages are achieved by the generation of a supersaturated solution, decrease of the particle size, improvement of wettability, or due to drug amorphization [123]. SDs can be structurally organized in two different ways, both containing two phases: as a solid crystalline dispersion, where the drug is in its crystalline form, or as an amorphous solid dispersion (ASD), where both drug and polymer are in the amorphous form. In the amorphous form, the drug is in a state of high energy, due to the lack of order in its chemical arrangement compared to its crystalline form. The higher molecular mobility of the amorphous drug increases the drug solubility and, consequently, the drug dissolution [37,124]. Therefore, considering its ability to promote an intrinsic interaction between drug and carrier, even at large scale and in a continuous manner, HME has been widely used in the development of SDs [64].

EE is the most used Eudragit in the goal to improve drug solubility by formulating ASDs, acting either as a polymeric matrix [50,51,63,66] or as a pH modifying agent [62]. This high demand is justified by the fact that EE has a good thermal stability and is a thermoplastic polymer, making it easier to process during HME due to its very low Tg. The preparation of theobromine SDs by HME using EE as the polymeric matrix afforded a better polymer-drug interaction, better powder flowability and drug dissolution properties compared with SDs prepared by freeze-drying and supercritical fluid [64]. In some cases, EE can act as a solubilizing agent, improving the aqueous solubility of BSC II drugs, like ibuprofen, felodipine and bifendate, from 12× to 300×, probably due to its ability to form micelles [63]. EE is also able to improve ibuprofen, indomethacin and naproxen solubility, even in the presence of high drug loading, thanks to the strong intermolecular interaction between EE and these drugs [69].

One of the most related uses of EE in the production of SDs and other formulations is as a taste-masking agent. The poor organoleptic properties of some drugs and the requirement of some specific groups, like paediatric patients, are the main reason for the use of this strategy in the development of oral delivery formulations [125]. EE has been largely used for this, due to its selective release properties., EE is insoluble at a pH above 5 and drug release from this polymeric matrix will therefore be avoided in the pH of the oral cavity (between 6.8 and 7.4) [27]. At the same time, in the gastric environment, EE is soluble and can release the drug immediately it is in contact with this medium. Isoniazid taste masking for paediatric administration was achieved by the formulation of extrudates containing EE. In vitro release studies performed in simulated salivary fluid showed that less than 1.55 mg mL^−1^ was released in this medium, whereas complete drug release was achieved in 0.1 N HCl after 45 min, independent of the drug loading (20% or 30%) [33].

Despite EE being the most used Eudragit polymer for the taste-masking of bitter drugs, EL100 and EL100-55 have also demonstrated this functionality. Melt-extrudates containing bitter drugs (cetirizine HCl and verapamil HCl) were produced with these polymers, and their efficacy on taste-masking was tested both in vivo in human voluntaries and in vitro using an Astree e-tongue system. The results demonstrated that both polymers were able to act as taste-masking agents, with EL100 having the advantage of dissolving in pH ≥ 6 compared with L100-55, which dissolves in pH ≥ 5.5 [80]. Similar findings were described for formulations prepared with these polymers and using propranolol HCl as the model drug [81].

The formulation of SDs can also overcome some other drug limitations relating to drug solubility behavior, as in the case of weak and sparingly soluble bases. These drugs are generally soluble in the gastric pH, but can precipitate in the area of absorption, as the intestinal environment has a neutral to basic pH. This behavior may result in a low oral bioavailability of these drugs. To overcome this drawback, enteric polymers can be used, which are able to release the drug only in the intestinal environment. EL100-55 is a good candidate for this purpose due to its pH-dependent solubility (soluble at pH > 5.5). This approach was used by Monschke (2019, 2021) in two sequential studies, in which nevirapine and ketoconazole were used as models of weak bases. In both cases, EL100-55 was combined with a plasticizer to improve its extrudability and was able to form an ASD, increasing the aqueous solubility of both drugs and avoiding their release in the gastric medium [73,75].

Despite the successful improvement of drug solubility by ASD production, in some cases, the amorphous forms may undergo a recrystallization process during dissolution, resulting in the precipitation of the dissolved drug. This is due to the formation of a super-saturated solution as a result of the rapid dissolution of the poorly water-soluble drug [63], which can directly affect drug release and, consequently, its oral bioavailability [50,55]. This effect can be overcome by using the correct concentration of polymer or polymeric blend, within the range that would be able to solubilize the drug. The Hansen solubility parameter (δ) has been used to predict miscibility between drug and polymer. Following this, when the solubility parameter between these two compounds is less than 7 MPa^1/2^, they are miscible, and when it is higher than 10 MPa^1/2^, they are not miscible. This miscibility between drug and polymer is highly related to the magnitude of their interaction [37,57,79,82]. In this context, some studies demonstrated that HME facilitates the interaction between the drug and the polymer. Maniruzzaman et al. (2013, 2015) evaluated the interaction between cationic drugs (propranolol HCl, diphenhydramine HCl, cetirizine HCl and verapamil HCl) and anionic polymers, showing that the amine functional group of the drug interacts by hydrogen bonds with the carboxylic group of some polymers, such as EL100 and EL100-55, which improves the solubility parameters between drug and polymer [79,82].

Besides the polymer:drug miscibility, another important point to be discussed is the polymer:drug ratio. The amount of polymer is closely related to the dissolution efficiency of a SD. Abu-Diak, Jones and Andrews (2011) used E4155F (freeze-dried EFS30D) to extrude ASD containing celecoxib as the drug. The higher the polymer:drug ratio, the greater the dissolution efficiency. In this case, E4155F was able to form a soluble complex with the drug, increasing the intrinsic drug solubility [77]. Similar behavior was presented in SDs prepared with osthole, a coumarin derivative, using EE as the polymer. The percentage of drug released in 30 min was 43%, 81% and 84% for drug:polymer ratios of 1:3, 1:6 and 1:9, respectively. This effect was mostly attributed to the drug crystallinity. In the 1:3 formulation, the drug was still present in its crystalline form, whereas there was a decrease in drug crystallinity in the 1:6 and 1:9 ratio formulations [55]. Eudragit polymers presenting pH-dependent solubility have also been used to formulate ASDs, depending on the site of action, or in other words, where the drug should be released from the polymeric material. EE is soluble at pH < 5 and has been used to promote gastric release [52,68], whereas E 4155F, which is soluble at pH > 7, has been used for colonic delivery, as the ionization of the free carboxylic acid groups occurs mainly at pH > 7 [77].

In most of the cases discussed above, the formulation of SDs by HME had the main goal of improving the solubility of poorly water-soluble drugs and accelerating drug release. However, it is also possible to improve the drug’s solubility and control its release through the rational choice of the polymer(s) and excipients [83]. ERL and ERS blends afford SDs with sustained drug release behaviour. Although ERL is able to control the drug release in some cases, the corresponding release profile may show a significant burst release phase due to its high permeability. On the other hand, ERS may result in an undesired controlled release of the drug over many hours. Whilst they have similar structures, ERL has a higher proportion of ammonium quaternary groups in its chemical structure than ERS, making the former more permeable [38]. However, the approach of using ERL and ERS blends has been reported as an interesting rational to obtain moderate burst release of the drug and faster sustained release, when compared with formulations composed of the single polymers [83]. Additionally, ERL:ERS mixtures were able to transform crystalline drugs and active substances, like curcumin, into their amorphous form and to enhance their bioavailability [83,84].

In other scenarios, the preparation of SDs may retard the release of highly water-soluble drugs, which can help to avoid some undesirable drug effects. For this purpose, ERS has been a good candidate, as it is a hydrophobic and insoluble polymer. Alshetaili et al. (2021) produced SDs with ERS to sustain the release of donepezil hydrochloride and, consequently, to avoid the side effects of its burst release. The physical interaction between the polymer and drug during the HME process (at 150 °C) was able to amorphise the drug, which improved its solubility, promoting a faster drug release in the first hour (about 20%), and a sustained release in the following 10 h [76]. In this specific case, the burst release was not suitable due to the drug’s characteristics, but ERS can be a good polymer candidate for drugs in which a burst release followed by a slow release is desirable.

Alongside the in vitro studies discussed above, the in vivo performance of SDs prepared with Eudragit polymers has also been demonstrated, mostly in terms of the oral bioavailability improvement of drugs. In a study performed by Zhang et al. (2014), SDs produced with EE were able to improve the *C_max_* of the flavonoid bacalein in beagle dogs by 2.68× after oral administration, resulting in better oral bioavailability compared to the pure drug [56]. The same behavior was presented for curcumin SDs prepared with a mixture of ERL and ERS. In rats, the relative bioavailability of curcumin was 223.44%, and the AUC_0-∞_ and *C_max_* of the SDs were higher than pure curcumin [84].

### 3.2. Tablets

After the production of SDs, some research groups added further steps to convert them into tablets [87,88,91,93,94]. As discussed in Section 3.1, SDs have been largely used as a strategy to improve the solubility of poorly soluble drugs. Additionally, this technological approach can overcome other drug limitations, such as their compression properties. For this purpose, extrudates are often milled into powders, mixed with other excipients and compressed. This process may or may not be successful. The materials used for HME and the compaction processes are decisive for the outcome. SDs of carbamazepine produced with EE by HME were able to transform the carbamazepine polymorph form III into form I, improving the drug wettability and, consequently, its dissolution rate. In addition, the compaction process after the HME improved the compactibility and tabletability of the powders, even at a concentration of 20% EE in the formulation [93]. Similar improvements in tabletability, compressibility and compactibility behavior for celecoxib-loaded SDs prepared with EE were described by Grymonpré and coworkers (2017) [91].

In fact, up to now, compression is still the most used technique to obtain tablets after HME, besides the growing use of HME processes to produce 3D printed solid forms, such as printlets and caplets. The classical process of wet granulation followed by direct compression was reported to produce nimodipine tablets after its extrusion with a mixture of EE and polyvinylpyrrolidone/vinyl acetate copolymer in different proportions. The tablets presented immediate drug release behaviour, despite the ratio of the polymeric blend [86]. In a different approach, Partheniadis et al. (2020) compressed extrudates produced with ERS or EL100-55, without any drug, at an ambient (20 °C) or elevated (40 °C) temperature. The use of hot compression was reported as an option to improve the tabletability of the materials after extrusion [96]. Besides compression, other techniques have been used to prepare tablets from formulations produced by HME. Injection moulding is a technique where a softened or melted material is injected under high-pressure conditions, allowing the production of objects with different shapes and sizes [126]. Because of these characteristics, HME coupled injection moulding has been used to prepare pharmaceutical forms. In this sense, ibuprofen and celecoxib biconvex tablets were obtained by using chemically modified EE [29,89]. The use of EE, even chemically modified, allowed immediate drug release tablets to be obtained.

As another versatile application of the Eudragit polymeric series, Patki and co-workers (2021) proposed an innovative system called the Overdose and Alcohol Sensitive Immediate Release System (OASIS), as tablets, to prevent overdose resulting from the exacerbated intake of sleeping tablets or the simultaneous intake of alcohol with sleeping tablets. Polymeric filaments containing an agonist (metoprolol tartrate) or an antagonist (hydrochlorothiazide) of GABA-A receptors (as model drugs) were produced by HME with EE or an ERL:ERS (7:3) blend, respectively. EE filaments were supplemented with an alkalizing agent. After extrusion, the two filaments were milled, and their powders mixed and compressed together into tablets. Therefore, if sleeping tablets were ingested in large quantities by an abusing patient, the alkalizing agent present in the EE filament could increase the gastric pH above 5. At pH above 5, EE is not soluble and the drug (metoprolol tartrate) would not be released by the tablet. Similarly, the mixture of ERS:ERL (7:3) was shown to be responsive to the presence of alcohol. Thus, if a patient took sleeping tablets accompanied by alcoholic drinks, the system would release the antagonist of GABA-A receptors (hydrochlorothiazide), and there would consequently be no therapeutic effect and no toxicity [7]. A similar system was developed by Nukala et al. (2019) to avoid the oral abuse of loperamide, an anti-diarrheal drug used to achieve euphoric effects. Filaments containing loperamide were prepared by HME, using EE as the polymeric material. The tablets were produced by compression of a powder mixture of the milled filaments and L-arginine, which was added as an alkalinizing agent. If these tablets were ingested in large amounts, the medium would be basified by the L-arginine, and the EE would not dissolve, avoiding release of the loperamide [92].

Orally disintegrating tablets (ODTs) are pharmaceutical dosage forms formulated to disintegrate almost immediately when in contact with saliva, even in the absence of water. However, depending on the drug, if it dissolves in the oral cavity, it can taste bitter or even irritate the local area, affecting treatment compliance and leading to undesirable effects. Therefore, it is imperative to look for alternatives to mask the taste of some drugs in order to facilitate the development of ODTs [127]. In this sense, EE has been widely used to mask the taste of bitter drugs whilst allowing immediate release into the gastric environment, due its solubility properties, as already discussed in Section 3.1. EE was used as a polymeric matrix to produce ibuprofen or mefenamic acid SD granules by HME, followed by their tabletting into ODTs. This approach allows taste masking of the drug, tested in vitro for mefenamic acid and in vivo for ibuprofen [85,88]. Going beyond the taste mask function, the production of EE SDs containing mefenamic acid was able to enhance the solubility of the drug by its amorphization. Moreover, mefenamic acid acted as a plasticizer in this formulation, which made the SD production easier, without the need to add an additional plasticizer [88].

The drug taste masking property of EE was also explored in the development of mini-tablets using the HME technique. Mini-tablets are solid systems with a diameter of 2–5 mm or smaller, having great appeal for children, geriatric patients and patients who have a general difficulty in swallowing. Mini-tablets were obtained by HME with the presence of a pelletizer at the end of the extruder [90,95]. EE was used as a polymeric matrix for the development of HME mini-tablets containing ketoprofen, to mask its bitter taste. The choice of EE in this formulation was also rationally based on the cationic behavior of this polymer, which facilitated the intermolecular interaction with ketoprofen, an anionic drug [90]. ERL was also used to develop acetaminophen floating mini-tablets, which were obtained by the injection of pressurized non-toxic and inert CO_2_ gas in different zones of the extruder, with the aim of forming pores in the polymeric matrix. ERL provides an adequate controlled drug release from mini-tablets over 3 h, as these systems remain floating in the gastric environment [95].

In fact, the conversion of HME extrudates into monolithic or multiple unit tablets with better acceptance among patients seems to be the most viable alternative to their use as SDs. As discussed above, polymers from the Eudragit family have been used to provide good properties for the compressibility of extrudates and to allow the modulation of drug release from the final dosage form. The different pH-independent and dependent solubility behaviors provided by this polymer family is probably the main reason for these versatile applications.

### 3.3. Other Dosage Forms Obtained by HME

Polymers from the Eudragit family have also been used to produce dosage forms by HME other than those discussed in the previous sections, such as ocular inserts [109], dry suspensions [105], or actually as extrudates [38,108]. These reports are summarized in Table 4, and the most relevant data is discussed below.

Among the innovative drug delivery systems that HME can produce are the transdermal films. Transdermal films can avoid first-pass metabolism, do not cause pain during administration and can be easily applied by the patient, improving both the success of the treatment and the patient adhesion [128,129]. Due it being biocompatible and already in use in transdermal applications, ERS was used as the polymeric matrix in the development of transdermal films containing ibuprofen, to overcome its gastrointestinal irritation by oral intake. HME equipped with a slit (sheet) die was used. The ibuprofen amorphization obtained by HME, combined with the hydrophilic agents added to the formulation (sucrose, methylcellulose, xanthan gum (Xantural175), poloxamer (Pluronic1F127) and Gelucire 44/14), led to an improvement of ibuprofen release, high hydration and permeation through silicone membranes, used to mimic the skin. Compared to the formulation prepared only with ERS, the formulation containing the hydrophilic additives were able to increase the ibuprofen permeation over 3 days, from 22% to 45%. The highest drug permeation (%) was achieved using 20% of Gelucire 44/14 as a hydrophilic excipient [11].

In the same way, oral films were also developed by HME using Eudragit polymers. Orodispersible films have the ability to dissolve in the mouth, even in the presence of a small volume of saliva. In this context, a blend of ERL with the water-soluble polymers poly (ethylene oxide) and hydroxypropyl methylcellulose were used to produce domperidone films, using PEG 3350 as plasticizer. The addition of ERL slowed down the domperidone release from the films (49.2% in 2 h) in comparison to those films composed only of the water-soluble polymers, poly (ethylene oxide), or a mixture of poly (ethylene oxide) and hydroxypropyl methylcellulose. These films released 67.3% and 82.7% of domperidone in 2 h, respectively. The authors explain that these different drug release profiles probably occur due to the differences in the solubility of the polymers, which can influence the swelling indices and erosion behavior of the films [112].

ERS orodispersible films were also produced by the association of HME and solvent casting methods to produce theophylline films with rapid disintegration time but prolonged drug release. First, extrudates containing the drug and ERS were produced by HME and milled in different sizes (<315 µm, 315–500 µm, 500–715 µm, 715–1000 µm and >1000 µm). In the next step, the milled powders (10% or 30% of drug) were mixed with hydroxypropyl methylcellulose (15%) and glycerol (6%) as plasticizer, in water, to produce the orodispersible films (20 × 30 mm) by the solvent casting method. These films showed a disintegration time of less than 180 s and a theophylline prolonged release was obtained at the same time. This prolonged release profile was reached using ERS as a polymer to produce the drug-load matrix particles, as well as using different size ranges of milled extrudates. This strategy was suggested by the author to avoid the risk of dose dumping by reducing the fluctuation in dissolution profile related to different gastrointestinal transit times [113].

Floating drug delivery systems are gastroretentive formulations used as a strategy to increase the gastric residence time of drugs. This strategy has been used to overcome the instability or low solubility of some drugs in the intestinal environment, as well as improve the bioavailability of drugs that are mostly absorbed in the upper gastrointestinal tract. ERS was used in the preparation of metoprolol succinate floating multiparticulates by HME. Sodium bicarbonate was added to the formulation before the extrusion process: once in the presence of an acid medium, gas is generated, enabling the matrix to float [117]. The same strategy was used by Vo et al. (2016) of produce foam strands. In this case, an injection of ethanol as foaming agent was performed during the HME process in the preparation of theophylline pellets. ERL and ERS were evaluated as the matrix polymers in the pre-formulation studies. ERS was chosen as the matrix former due to its better strand formation, floating ability and dissolution properties of the pellets [121]. An alternative approach to produce floating formulations by HME was developed by Simons and Wagner (2019). First, EE and ERS were blended with metformin and stearyl alcohol as a plasticizer. During the HME process, the extruder was equipped with a modular design tube d to form hollow tubes. After the extrusion process, the ends of the hollow tubes, which contained metformin in their walls, were sealed with a heated circular cavity. This strategy was reported to be suitable for the development of a high drug loading (50% to 80%) formulation, with sustained release, without any burst release and independent of the EE:ERS ratio [120].

Pellets can be obtained as drug delivery systems after the HME process, by strand pelletizer [9,107,118], die face pelletizer [110], or even by cutting manually [99,104]. They have been produced by HME with two main objectives: (a) as a drug delivery system itself, or (b) as a stock material for the production of another pharmaceutical form, such as tablets [107], as previously discussed in Section 3.2. In that regard, EE was used as a strategy to provide rifampicin immediate release, as an attack dose, whereas hydroxylpropyl cellulose was used as the extended release polymer for dose maintenance [99]. Furthermore, ES100 was also reported as an enteric polymer for the development of chronotherapeutic ibuprofen and ketoprofen pellets by HME, prepared with different sizes (1, 2 and 3 mm). ES100 and ethyl cellulose, a hydrophobic polymer, were blended before the extrusion process, resulting in a drug release profile from the pellets with a lag time of about 6 h (in 0.1 N HCl, 2 h + pH 6.8, 4 h). After this time, changing the release medium to pH 7.4, the release of both drugs showed a sustained profile from pellets, with the drug release amounts being influenced by the size of the pellet and by the concentration of ethyl cellulose (0%, 2.5%, 5% and 10%) [9].

Besides all the studies regarding the development of delivery systems composed of Eudragit polymers by HME, as discussed in the previous sections, the coupling of HME to 3D printing has been widely explored by many research groups in pharmaceutics in recent years. In this scenario, polymers from the Eudragit family have been gaining attention as matrix polymers in the development of 3D printed pharmaceuticals, as presented and discussed in the next section.

## 4. 3D Printing

Researchers have paid important attention to the versatility of 3D printing to produce pharmaceutical dosage forms with customized dose, size, shape, color, and release profile. In this scenario, the use of the Eudragit polymer family in the 3D printing of pharmaceuticals has grown markedly in the last 7 years. Forty of the 122 articles from our data survey, representing 32.8%, were dedicated to this area. Among them, 87.5% reports used at least one Eudragit polymer to prepare 3D printed dosage forms by FDM (Table 5), whereas 12.5% used other 3D printing techniques, like direct extrusion and SLS (Table 6).

### 4.1. Fused Deposition Modeling

In FDM 3D printing, some factors are able to influence the quality of the printed object and even decide if the material is printable or not [168]. Among them, an important factor is the feedability. A feedable filament must have adequate mechanical properties to ensure that it passes through the printer’s feeding and heating zone, so that it would be ready for printing itself. Some authors indicate these three reasons why a filament may not be suitable for 3D printing: feeding gears can break filaments that are too brittle; the nozzle cannot push soft filaments; and the feed gears can scratch filaments that do not have enough stiffness [149]. A too brittle or too soft filament can break inside the feeding zone, obstructing the passage, and is unsuitable for 3D printing [169]. Sometimes, polymers alone are not able to provide good feedability because they are not plastic enough. This is the case with EE. In a study by Nasereddin et al. (2018), EE filaments (100% *w*/*w*), without drug, did not present adequate feedability because they were too brittle. Alternatively, other excipients were successfully added as options to improve the feedability of the filaments, e.g., Tween 80, polyethylene glycol 4000 and polyethylene oxide, in concentrations of 11%, 16.7% and 16.7%, respectively [133]. Indeed, filaments with good feedability are essential for FDM 3D printing, and this feedability is assessed by adequate mechanical properties, like flexibility and filaments that are not too brittle. Therefore, for polymers that are not too plastic, like some members of the Eudragit family (EE, ERS, and ERL), excipients that offer these characteristics to the formulation, such as plasticizers and lubricants, are of interest and have been extensively used due to their ability to improve melt viscosity and polymer plasticization [130].

Additionally, the filament may be feedable but not printable in some cases. The reasons for this problem are similar to those explained above for feedability: inadequate rheological and mechanical properties. In this context, texture analysis has been an important ally in determining the printability of the filaments. In a study performed by Xu et al. (2020), the three-point bend test, resistance test and stiffness test were responsible for providing the brittleness, resistance value and toughness data, respectively. As a result, toughness was the only parameter that showed good correlation between printable and not printable filaments. For the printer used in this study, a toughness of 80 kg/mm^2^% was the lowest value required for a printable filament. For example, filaments composed of ERL and indomethacin (70:30 *w*/*w* %), without any plasticizer, presented a toughness of 18.4 kg/mm^2^% and were not printable [149]. In cases where Eudragit was not printable, this issue was solved by adding a plasticizer to the formulation, or even using polymeric blends [157], as will be further discussed in more detail below.

After overcoming the feedability limitation, Eudragit has been used with different functions in FDM 3D printing, especially as the main polymeric matrix of tablets, printlets or caplets. Due to its chemical characteristics, EE has been the most used in the development of 3D printed immediate drug release formulations. To improve the printability of EE filaments, mixtures with other polymers like hydroxypropyl cellulose [139], polyethylene oxide [136] and excipients like plasticizers [132,138] have been reported. Hydroxypropyl cellulose is an easily extrudable polymer, once its melt viscosity decreases during heating in the 3D printing process. Good results have been shown when it is mixed with EE, in concentrations ranging from 30% to 35% of EE and 5% to 20% of hydroxypropyl cellulose. These filaments had adequate mechanical properties and are easy printability, providing immediate theophylline release after 3D printing as tablets [139]. Moreover, the addition of plasticizers, like triethyl citrate, was able to decrease the T_g_ of the EE in filaments containing warfarin from 500 µg to 2500 µg, making their printing easier [132].

Sadia and co-authors (2016, 2018) used EE as the main polymer in the development of two innovative platforms of oral drug delivery. In the first one, EE’s ability to produce immediate release caplets containing 4 drugs with different physicochemical characteristics was established. For this purpose, beyond the use of triethyl citrate as plasticizer, tri-calcium phosphate was added to the tablet formulation as a non-melting filler, showing an important role in the roughness of the filament and consequently improving printability. Independent on the acidic (5-ASA and captopril), basic (theophylline) and neutral (prednisolone) property of the drug model, the drugs were completely released from the caplets after 30 min, showing the suitability of using EE in the production of 3D printed pharmaceutical forms with immediate drug release behaviours [131]. In a subsequent study, bilayer antihypertensive tablets containing enalapril maleate and hydrochlorothiazide were printed and had their dose controlled by the thickness of the tablet layer. Both drugs presented immediate release profiles, explained by the release mechanism from the EE matrix, which was controlled by the erosion of the methacrylate polymer. Immediate release occurred despite the differences in drug solubility in aqueous medium and their crystallinity pattern in the polymeric matrix – amorphous enalapril and crystalline hydrochlorothiazide [135]. On the other hand, these tablets had low friability due to the high content of EE in their structure (more than 46%). Moreover, EE can be used as a taste masking agent, as already discussed in Section 3.1, due to its ability to remain unchanged in the neutral pH of the mouth, protecting the drug from the patient’s taste buds, but releasing the active ingredient immediately it is in contact with the gastric medium [1].

ERL is characterized as a polymer with swelling properties but no erosion properties in aqueous medium, affording a gel formation [148]. For this reason, it has been used as an option in the formulation of sustained drug release 3D printed tablets [147,148,160] and dual-release systems, combining immediate and prolonged drug release in blends with polyvinyl alcohol [146]. Blends between ERL and polymers that are not so flexible, e.g., hydroxypropyl cellulose, can facilitate the printability of these systems. Tan, Maniruzzaman and Nokhodchi (2020) showed that, in mixtures of ERL, hydroxypropyl cellulose, polyethylene glycol and theophylline at different proportions (*w*:*w*), the presence of ERL in the formulation was responsible for the plasticity and smoothness of the produced filaments, ensuring their printability [34].

Another innovative proposal was reported by Beck and co-workers (2017), comprising the alliance of nanotechnology and 3D printing to produce nanomedicines. In this approach, ERL100 was used to prepare the polymeric filaments, which were 3D printed as tablets. Deflazacort-loaded nanocapsules were loaded in the tablets by a passive method, where the tablets were soaked in the nanocapsules suspension. The swelling properties of ERL100 provided a high drug loading compared to a non-swellable polymer (poly-epsilon-caprolactone). The drug release from the ERL depended on the presence of a pore former and the infill percentage [150]. Earlier, in 2015, ERL100 was used to print theophylline tablets with different sizes, which implies doses ranging from 60 mg to 300 mg, with a dose accuracy between 91% and 96%. In addition, these data corroborated with previous studies showing the importance of the addition of a high melting point (273 °C) component, in this case the water-soluble drug theophylline, to the methacrylic filaments, to improve their flow thought the nozzle, consequently improving printability. This system showed extended drug release over 16 h, governed mainly by the drug diffusion from the ERL100 matrix. In the same study, the mixture of ERL100 with ERS100 (1:1) slowed down the drug release rate, which was explained by the lower percentage of quaternary ammonium groups of ERS, making its structure less hydrophilic, and consequently less permeable [156].

On the other hand, ERS was used as the main polymer by Krause et al. (2019) in the 3D printing of an acetaminophen innovative pressure-controlled drug delivery system. This polymer was selected due to its insolubility in water, pH independent swelling properties and low permeability. In addition, its choice was also based on the ability of ERS100 to produce brittle capsule shells that would break in pressure conditions, affording immediate drug release under physiological conditions in the gastric environment [155]. Similarly to ERL, ERS has been also used in the 3D printing of controlled/sustained drug release formulations, like implants [151], or in blends with other polymer families [152,153]. Ilyés et al. (2019a) evaluated a mixture of ERS and hydroxypropyl methylcellulose (Affinisol HME 15LV) in the formulation of gastro-retentive 3D printed carvedilol tablets. The use of ERS in this study prolonged the gastric residence of the tablets, increasing their acid-resistance. Alternatively, ERS (in concentrations ranging from 38% to 62%) was used in a mixture with polyvinylpyrrolidone (25–49%) in the development of 3D printed skin patches containing a poorly soluble drug, quercetin. The mixture of ERS with a hydrophilic polymer, such as polyvinylpyrrolidone, provides a sustained drug release over 72 h due to the formation of a rigid matrix by the mixture of these two polymers. This approach afforded a reduced fluctuation of quercetin levels in rats’ plasma after the application of the 3D printed skin patches [153].

Among the polymers comprising the Eudragit family, EL100-55 and EL100 have been used as delayed release polymers in the 3D printing of drug delivery systems, either alone or in blends with other polymers, such as ethyl cellulose N14 [142] and hydroxypropyl cellulose EF [157]. In this context, EL100-55, which dissolves at pH above 5.5, has been used for delivery of drugs in the upper bowel, specifically in the duodenum, whereas EL100 is targeted for delivery in the jejunum, dissolving at pH above 6.0. Aiming to avoid the gastric irritation of aspirin, administered together with simvastatin, EL100-55 was used for the preparation of filaments that were later printed in the form of a two compartment polypill. EL100-55 filaments produced by HME were used to print the body of the polypills, with two compartments to avoid contact between the two drugs due to their incompatibility. The compartments were filled with molten polyethylene glycol 6000 mixed with glycerin, silica and aspirin or simvastatin. The blend was made by the melt casting technique, which was then directly injected into the 3D printed pill compartment. This association between 3D printing and the melt casting method was able to produce a single dosage form with two incompatible drugs [145]. EL100-55 was also used in a mixture with ES100 to target 5-fluorouacil alginate beads to the colon. This polymer helped to provide a colonic 5-fluorouacil release from hollow pH-responsive 3D printed tablets, which were produced in two layers: the upper layer with polylactic acid and the lower layer with Eudragit. The tablets were produced with an infill of 30%, which allowed distribution of the beads into the hollow area during the printing process. In gastric conditions, the Eudragit layer was not soluble, so the beads were not released from the tablets; whereas in the colon, the progressive erosion of Eudragit could release the beads containing the drug [161]. These two examples above, showed that, in addition to being the carrier of the drug itself, Eudragit polymers can be used as a strategy for the development of multiparticulate systems that carry other dosage forms, preventing dose dumping or the release of drug to unwanted sites.

It is important to note that Eudragit polymers can not only be used as a main component of the polymeric matrix but can also act as release modifiers and even as coating materials. Shi et al. (2021) printed ibuprofen tablets by FDM using ethyl cellulose as the main polymer of the filaments, blended with other polymers (poly(vinyl alcohol), hydroxypropyl methylcellulose, ERL, ERS), which were added as release modifier excipients. In this context, the incorporation of ERL (20%) or ERS (20% and 10%) to these filaments and tablets resulted in a decrease in the drug release rate from tablets compared with those tablets prepared with other polymeric release modifiers. In addition, the ibuprofen release rate from tablets containing ERL was slightly superior to those containing ERS, which is explained by the higher permeability of ERL to aqueous media compared with ERS, due to differences in the proportion of ionizable cationic groups, as previously discussed. In another study, Melocchi et al. (2019) used a mixture of ERS and ERL (1:1) to coat alopurinol poly(vinyl alcohol) 3D printed drug delivery systems for gastric retention, prolonging the duration of the drug release from these systems for 6 h, as expected, while the uncoated systems released close to 100% of the drug in 2 h [162].

Some types of Eudragit polymers claim special attention due to their ability to delay the release of the drug in the gastric environment, conferring enteral release. An enteral polymer must be resistant to gastric acid pH and release the drug in enteral conditions. EL100-55 is a methacrylic acid-ethyl acrylate copolymer that responds to pH stimulus, being insoluble at physiological pH and soluble in pH higher than 5.5. Due to its chemical structure and characteristics, it has been largely used as an enteric polymer [143]. Okwuosa et al. (2017) printed core-shell tablets by FDM for the delayed released of three different model drugs (theophylline, budesonide and diclofenac sodium). Polyvinylpyrrolidone filaments were used to prepare the core of the 3D printed tablets, whereas EL100-55 filaments were used to produce the shell. The use of EL100-55 in a shell thickness ≥0.52 avoided the drug release in the acid medium [143]. Moreover, EL100-55 was also used as an adjuvant polymer in a mixture with polylactic acid to obtain filaments by HME, as a way of providing enteric release profiles for 3D printed capsules of riboflavine-5′-phosphate [144].

Lastly, another strategy reported to provide an enteric release property for 3D printed tablets has been demonstrated by their coating with EL100. 3D forms containing budesonide printed from poly(vinyl alcohol) filaments were coated with a EL100 dispersion in isopropanol and water, using a spray fluidized bed coater [141]. Also, theophylline 3D printed pharmaceutical forms were prepared and coated with a EFS30D dispersion in the top layer [140]. Using these strategies, the drugs were only released at the intestinal pH, make them suitable alternatives to promote gastric mucosa protection against the irritation effects of the drugs.

### 4.2. Other Techniques

According According to the studies discussed in the previous sections, Eudragit polymers have been widely explored in recent years to tailor the drug release rate from 3D printed forms prepared mainly by the FDM technique. However, some variations of this technique have been proposed, and this polymer family continues to have an important role in these new platforms. Variations in the FDM technique include the exclusion of a previous HME process to prepare the filament, as an intermediary product. This new 3D printing techniques has been called the direct extrusion technique and consists of a single step technique where the thermal extrusion of powders, blends or pellets is achieved by the use of pneumatic or screw-based extrusion at high temperatures during the feeding of the 3D printer [165]. ERL and ERS, alone or in a mixture, were reported in the formulation of theophylline 3D printed tablets, which also contained glyceryl monostearate, acting as plasticizer and lubricant, and triethyl citrate, as plasticizer. The drug release from the 3D printed tablets could be modulated by the variation of the ERL:ERS ratio in the formulation: the higher the content of ERS, the slower the drug release from the 3D printed dosage forms [165]. The application of EE was also demonstrated in direct extrusion 3D printing in a recent study. In this case, a poorly-soluble drug, dutasteride, was selected as a model drug in the printing of dosage forms with four different geometries (cube, pyramid, hemisphere and tube). The printing process was able to produce amorphized dutasteride; however, unexpected data were observed in the drug release studies. The drug was not released from the dosage forms in either acidic or neutral pH, probably because the strong drug:polymer interaction made ionization of the polymer impossible, which is a phenomenon that allows EE solubilization at pH conditions below 5 [163].

The use of Eudragit polymers has also been reported in the 3D printing of pharmaceuticals using the SLS technique. Fina et al. (2017, 2018) used EL100-55 and a blend of EL100-55 and ERL to print medicines by SLS. EL100-55 was used as a modified release polymer in the 3D printing of paracetamol printlets, in different polymer:drug ratios (92:5; 77:20; 62:35). Due to the inability of the EL100-55 to be sintered by the laser light, as it does not absorb laser light in the wavelength used (445 nm), 3% Candurin^®^ was used as an excipient. Printlets were successfully printed, independent of the drug loading and with no drug degradation, presenting a delayed release, independent of their drug percentage [166]. The same strategy was also explored with Candurin^®^ in a subsequent study, where EL100-55 and ERL were used, independently, as model polymers in the preparation of paracetamol printlets with cylindrical and gyroid lattice structure. The cylindrical printlets, mostly produced with ERL, were able to delay the drug release, whereas the gyroid structure released about 70% in 2 h (EL100-55) and 100% in 2 h (ERL), probably because of the high porosity of this system, which increases the surface area [167]. According to the studies described above, both Eudragit polymers are suitable for the 3D printing of pharmaceutical dosage forms by SLS.

## 5. Quality Assessment of Dosage Forms Produced by HME and 3D Printing

Regarding the characterization and quality assessment of dosage forms produced by HME and 3D printing techniques, a common set of techniques were identified in the reports comprised here. Although the main discussions in this review were based on the use of Eudragit polymers in the tailoring of drug release properties from solid forms produced by HME and 3D printing processes, it is important to highlight the most relevant techniques that have been used to characterize them and to understand the effects of changes in the process or components of the formulations on their properties and behaviours. Among the most relevant techniques, thermal analysis, X-ray powder diffraction (XRPD) and infrared spectroscopy (IR) can be cited. Thermogravimetric analysis (TGA) and differential scanning calorimetry (DSC) are thermal characterization techniques used to determine the thermal stability of products [170]. From TGA data, it is possible to determine the temperature range in which drugs and excipients degrade, and thereby establish the best temperature to be used in the extrusion and thermal printing processes [163]. As a general rule, to produce adequate flow through the extruder, the work temperature has to be set above the polymer T_g_, reducing the viscosity of the material but avoiding drug thermal degradation [74]. The use of proper temperatures favours the production of extrudates and/or 3D printed forms, and the amorphization of the drug. Drugs with high melting point have greater difficulty in amorphization due to the greater energy required for this conversion [66].

DSC and XRPD are complementary techniques in the evaluation of the drug crystallinity state [137]. Generally, drugs in the crystalline state are more stable and less soluble. When submitted to thermal processing, the drug can be solubilized by the molten polymer and go from its crystalline to the amorphous form, which is more chemically and physically unstable, but more soluble, when compared to its crystalline state [72]. Besides the physical state, DSC analysis can also be used to determine the phase transition, miscibility and stability of a thermodynamic system [78]. On the other hand, IR has been extensively used to determine the drug-polymer interaction in products obtained by extrusion and 3D printing, and also to evaluate possible degradation during the production process [64]. The appearance and disappearance of IR bands can indicate a molecular interaction [57,61], while no change reflects no interaction [117].

Furthermore, image techniques like scanning electron microscopy (SEM) and X-ray micro-computed tomography (µCT) have been used to characterize the dosage forms by HME and 3D printing, both micro and macroscopically. SEM has been used to evaluate the surface morphology [9,161], cross-sectional morphology [145] and also to observe the integrity of structures present in the formulation [150] in both filaments and 3D printed products. µCT is a non-destructive image technique that has been used to observe the inner structure of the formulations [134], making it possible to calculate the surface area, solid volume and open/close pores volume of formulations [171]. Confocal Raman microscopy is an innovative and highly sensitive technique that allows the identification of information about the components in the structure, using in tin samples [172]. In dosage forms produced by HME and 3D printing, confocal Raman has also been used to identify the crystallinity state of molecules [54,171] and to evaluate the spatial distribution of the components in the formulation [150].

## 6. Final Remarks and Challenges

The use of plasticizers is a trend in the production of drug delivery systems by HME and 3D printing, where its addition to the formulation can decrease the T_g_ and the processing temperature of the polymers, helping in the maintenance of drug stability [96]. According to the reports discussed in this review, examples of this type of excipient are triethyl citrate [111,134,146], propylene glycol [109], citric acid monohydrate and Lutrol F127 [115], stearic acid, glyceryl behenate and polyethylene glycol 8000 [103], polyethylene glycol 2000, Poloxamer 188, and Cremophor RH [56]. In some cases, the drug itself can act as a plasticizer, as reported for ibuprofen [11,74], naproxen [111] and mefenamic acid [88].

Another approach in the development of drug delivery systems with Eudragit polymers that should be highlighted is the use of blends with polymers of different classes and with specific properties. These blends can be used to modulate the drug release, improve the flow properties and assure the stability of the formulations [52,62,133,143]. Due to the high number of available polymers for pharmaceutical use, the study of blends with Eudragits can be considered a field to be explored in the development of new drug delivery systems by HME and 3D printing.

In fact, the use of plasticizers in the production of formulations by HME and 3D printing has been widely applied, even posing a challenge for formulators to develop a basic formulation without any additives. This dependency is even more pronounced in 3D printing, when Eudragit printable filaments have rarely been achieved without the presence of plasticizers or lubricants. Although the use of plasticizers has helped to reduce working temperatures, drugs that degrade at high temperatures can often still not be formulated by FDM, since some Eudragits have a high T_g_, especially those used to produce a delayed release (EL100, ES100 and EL100-55). In addition, even when drugs can be added to these formulations without undergoing thermal degradation, it has still been a challenge to transform them into their amorphous form, which depends on the drug’s melting point.

Other challenges faced by the production and clinical translation of drug delivery systems by 3D printing are the lack of information about regulatory issues, and further pharmacokinetic studies and clinical trials with the innovative formulations. Up to now, in vivo trials of formulations composed of Eudragit polymers and prepared by HME and 3D printing comprise pre-clinical trials using animals like beagle dogs [151], and the evaluation of taste masking properties of these formulations in human volunteers [62]. This is a challenge not exclusively faced by Eudragit polymers, but in general, owing to the recent application of this technique in pharmaceutics. Nevertheless, the continuous efforts and the growing number of papers devoted to producing pharmaceuticals with Eudragit polymers is capable of promptly surpassing these challenges.

## 7. Conclusions

This review provides an overview of the applications of Eudragit polymers in the development of drug delivery systems by HME and 3D printing reported in the last ten years. The different properties attributed to the different grades of the Eudragit polymers make them versatile ingredients to obtain dosage forms by these methods, ranging from immediate to controlled drug release systems. Studies have demonstrated strategies to overcome some limitations that could impair the use of these polymers in these processes. The use of plasticizers, the combination of different Eudragit polymers or even blends with other polymers and materials have been reported among these strategies. More recently, the coupling of HME and 3D printing techniques has paved the way to the development of innovative pharmaceuticals intended for the customization of therapies. The scientific scenario presented here offers a range of opportunities to be explored in pharmaceutics using well-known GRAS excipients, such as the Eudragit polymers, and advanced manufacturing processes, like 3D printing, to boost the clinical translation of personalized medicines.

## Figures and Tables

**Table 1:** Different Eudragit grades, their chemical composition, and physical properties.

Eudragit	Substituents	pH-Dependent Solubility	Molecular Weight (g/mol)	Glass Transition Temperature (°C)	Availability
Eudragit E PO	R_1_, R_3_ = CH_3_, R_2_ = CH_2_CH_2_N(CH_3_)_2_, R_4_ = CH_3_, C_4_H_9_	Gastric fluid up pH 5.0	47,000	48	Powder with amine like odor
Eudragit E 100	R_1_, R_3_ = CH_3_, R_2_ = CH_2_CH_2_N(CH_3_)_2_, R_4_ = CH_3_, C_4_H_9_	Gastric fluid up pH 5.0	47,000	48	Granules
Eudragit RL PO	R_1_ = H, CH_3_, R_2_ = CH_3_, C_2_H_5_, R_3_ = CH_3_, R_4_ = CH_2_CH_2_N(CH_3_)_3_^+^ Cl^−^	Insoluble, high permeability	32,000	70	White powder with a faint amine like odor
Eudragit RL100	R_1_ = H, CH_3_, R_2_ = CH_3_, C_2_H_5_, R_3_ = CH_3_, R_4_ = CH_2_CH_2_N(CH_3_)_3_^+^ Cl^−^	Insoluble, high permeability	32,000	70	Colorless, clear to cloudy granules with a faint amine like odor
Eudragit RS PO	R_1_ = H, CH_3_, R_2_ = CH_3_, C_2_H_5_, R_3_ = CH_3_, R_4_ = CH_2_CH_2_N(CH_3_)_3_^+^ Cl^−^	Insoluble, low permeability	32,000	64	White powder with a faint amine like odor
Eudragit RS100	R_1_ = H, CH_3_, R_2_ = CH_3_, C_2_H_5_, R_3_ = CH_3_, R_4_ = CH_2_CH_2_N(CH_3_)_3_^+^ Cl^−^	Insoluble, low permeability	32,000	64	Colorless granule with a faint amine like odor
Eudragit L100	R_1_, R_3_ = CH_3_, R_2_ = H, R_4_ = CH_3_	Above pH 6.0	125,000	150	Solid powder with a faint characteristic odor
Eudragit L100-55	R_1_, R_3_ = H, CH_3_, R_2_ = H, R_4_ = CH_3_, C_2_H_5_	Above pH 5.5	320,000	110	White powder with a faint characteristic odor
Eudragit S100	R_1_, R_3_ = CH_3_, R_2_ = H, R_4_ = CH_3_	Above pH 7.0	125,000	150	White powder with a faint characteristic odor
Eudragit FS 30D	R_1_ = H, R_2_ = H, CH_3_, R_3_ = CH_3_, R_4_ = CH_3_	Above pH 7.0	280,000	48	Aqueous dispersion 30%, Milky-white liquid of low viscosity with a faint characteristic odor

References: [26,27,28].

**Table 2:** Solid dispersions produced by HME using Eudragit polymers.

Eudragit Type	Extrusion Temperature (°C)	Polymer Role	Drug	Release Data ^$^	Reference
Eudragit E	130	Polymeric matrix	Fenofibrate	≅70% in 90′ (drug:polymer); ≅100%, 15′ (drug:polymer:MA)	[49]
Eudragit E PO	180	Polymeric matrix	Bifendate	≅90% in 30′	[50]
	120	Polymeric matrix	Efavirenz	96% in 30′	[51]
	165 and 185	Polymeric matrix	Carbamazepine	100% in 20′	[52]
	150	Polymeric matrix	Felodipine	≅37% in 40′ (10% drug); ≅11% in 40′ (30% drug); ≅12% in 40′ (50% drug); ≅15% in 40′ (70% drug)	[53]
	110–150	Polymeric matrix	Spironolactone	> 95%, 60′	[54]
	85	Polymeric matrix	Osthole	43% in 30′ (drug: polymer, 1:3), 81% in 30′ (drug: polymer, 1:6); and 84% in 30′ (drug: polymer, 1:9)	[55]
	160	Polymeric matrix	Baicalein	90%, 90′	[56]
	5 °C higher than the melting point of the individual drugs	Polymeric matrix	Carbamazepine, celecoxib, felodipine, fenofibrate	*	[57]
	110	Polymeric matrix	Piperine	≅20% in 120′	[58]
	110–150	Polymeric matrix	Felodipine	*	[59]
	170 ^#^	Polymeric matrix	Itraconazole	*	[60]
	90	Polymeric matrix	Ibuprofen	85% in 5′	[61]
	110–140	pH modification agent	Meloxican	*	[62]
	130	Polymeric matrix/Taste-masker agent	Isoniazid	100% in 5′ (20% drug); 100% in 15′ (30% drug)	[33]
	90–180	Polymeric matrix	Bifendate, felodipine and ibuprofen	100% in 15′ (1% BIF); 100% in 15′ (0.7% FEL); >90% in 15′ (1.5% FEL); 100% in 10′ (4% IBU); 100% in 10′ (10% IBU);	[63]
	150	Polymeric matrix	Theobromine	>80% in 10′	[64]
	150–160	Polymeric matrix	Cocoa extract/Theobromine	≅80% in 30′ (EPO); ≅86% in 30′(EPO: Sol); ≅85% in 30′ (EPO:PVP); ≅80% in 30′ (EPO:Sol:PVP)	[65]
	160	Polymeric matrix	Resveratrol	≅85% in 20′	[66]
	80–150	Polymeric matrix	Mesalamine	>97% in 60′	[67]
	160	Polymeric matrix	Indomethacin	≅84% in 15′	[68]
	65–120	Polymeric matrix	Ibuprofen, indhometacin and naproxen	*	[69]
	140	Polymeric matrix	Indomethacin	≅54% in 5′ (drug:EPO, 4:1); ≅28% in 5′(drug:EPO: PVP, 4:1:0.01); <22% in 60′ (drug:EPO: PVP, 4:1:0.05 and 4:1:1)	[70]
Eudragit L100-55	160	Polymeric matrix	Itraconazole	18% in 2 h	[71]
	130	Polymeric matrix	Lumefantrine	*	[72]
	100–150	Polymeric matrix	Nevirapine	<5% in pH 1 (milled and pellet); ≅30% (milled) and ≅10% (pellet) in pH 5.5; ≅90% (milled) and ≅70% (pellet) in pH 6.8	[73]
	90–170	Polymeric matrix	Ibuprofen	≅90% in 60′, PBS pH 6.8	[74]
	100–140	Polymeric matrix	Ketoconazole	**	[75]
Eudragit RS PO	150	Polymeric matrix	Donepezil hydrochloride	≅30% in 10 h	[76]
Eudragit 4155F	170	Polymeric matrix	Celecoxib	100.67% in 72 h (drug:polymer, 1:9); 53.37% in 72 h (drug:polymer, 3:7)	[77]
Eudragit E PO	***	Polymeric matrix	Indomethacin, itraconazole and griseofulvin	109.8% in SGF (IND:polymer, 30:70)	[78]
Eudragit L100				*	
Eudragit L100-55				74.5% in SIF (IND:polymer, 30:70); 1.9% in SGF and 20.1% in SIF (ITZ:polymer, 30:70); 94.7% in SIF (GSF: polymer, 30:70)	
Eudragit L100	130–165	Polymeric matrix	Propranolol HCl and diphenhydramine HCl	*	[79]
Eudragit L100-55	100–115				
Eudragit L100	100–155	Polymeric matrix/Taste-masker agent	Cetirizine HCl and verapamil HCl	>70% in 2 h (cetirizine); >80% in 2 h (verapamil)	[80]
Eudragit L100-55					
Eudragit L100	100–155	Polymeric matrix/Taste-masker agent	Propranolol	*	[81]
Eudragit L100-55					
Eudragit L100	100–155	Polymeric matrix	Cetirizine HCl and verapamil HCl	*	[82]
Eudragit L100-55					
Eudragit RL PO	90–140	Polymeric matrix	Metropolol	*	[37]
Eudragit RS PO					
Eudragit RL PO/RS PO	135–150	Polymeric matrix	Curcumin	≅90% in 12 h	[83]
	135–150	Polymeric matrix	Curcumin	Varying between ≅70% and >90% in 12 h, depending on the extrusion temperature, screw speed, cooling rate and particle size.	[84]

* release studies were not performed; ** release studies perfomed in two different media, with 6 different particle size, and two different drug loads; *** not clearly identified in the method; ^#^ study used EL100, EL100-55, ES100, ERS, ERL, EFS30D and ENE 30D for a theoretical screening, but only EE was effectively extruded; ^$^ detailed release data are shown due to the lack of information in some original studies impairing the classification of the drug release behavior (immediate, controlled, or delayed). BIF, Bifendate; FEL, felodipine; GSF, griseofulvin; IBU, ibuprofen; IND, indomethacin; ITZ, itraconazole; MA, malic acid; PBS, phosphate buffer solution; PVP, poly(vinylpyrrolidone-co-vinylacetate); SGF, simulated gastric fluid; SIF, simulated intestinal fluid; Sol, Soluplus^®^.

**Table 3:** Tablets produced from HME products prepared with Eudragit polymers.

Eudragit Type	Extrusion Temperature (°C)	Polymer Role	Drug	Release Data ^$^	Technique of Tablets Obtantion	Reference
Eudragit E PO	140	Polymeric matrix	Ibuprofen	≅65% in 120′ (drug: 25%); ≅95% in 120′ (drug:40%)	Compression	[85]
	80–130	Polymeric matrix	Nimodipine	80% in 10′	Compression	[86]
	90–125	Polymeric matrix	Naproxen	73% in 12 h, and 100% in 24 h (98.5% polyelectrolyte complex); 80% in 2 h (70% polyelectrolyte complex)	Compression	[87]
	100–120	Polymeric matrix	Ibuprofen and celecoxib	≅100% in 15′ (pH 1) and in 60′ (pH 3)	Injection molding	[29]
	110	Polymeric matrix	Mefenamic acid	>80% in 5′	Compression	[88]
	90	Polymeric matrix	Ibuprofen	≅100% in 20′ (pH 1); ≅100% in 40′ (pH 3); <10% in 60′ (pH 5 and 7)	Injection molding	[89]
	100–120	Polymeric matrix	Ketoprofen	100% in 20′	Pelletization	[90]
	120–140	Polymeric matrix	Celecoxib	*	Compression	[91]
	150	Polymeric matrix	Loperamide	>85% in 15′ (single unit); <2% in 45′ (multiple unit)	Compression	[92]
	140	Polymeric matrix	Carbamazepine	>85% in 10′ (drug:polymer, 2:1 and 4:1); >85% in 20′ (drug:polymer, 1:1)	Compression	[93]
Eudragit L100-55	170–180	Polymeric matrix	Griseofulvin	<5% in pH 1.2; ≅36% in pH 6.8 (drug:polymer:TEC); ≅42% in pH 6.8 (drug:polymer:K12:TEC); ≅60% in pH 6.8 (drug:polymer:S630:TEC); ≅66% in pH 6.8 (drug:polymer:S630:ATBC, <45 μm); ≅66% in pH 6.8 (drug:polymer:S630:ATBC, <250 μm);	Compression	[94]
Eudragit RL PO	90–165	Polymeric matrix	Acetaminophen	86.5% in 3 h	Pelletization	[95]
Eudragit E PO/RL PO/RS PO	150	Polymeric matrix	Metoprolol tartrate and hydrochlorothiazide	MT = 100% in 60′ (FaSSGF); <60% in 30′ (FaSSGF + 20% ethanol); HCT ≤ 20% in 30′ (FaSSGF); >50% in 30′ (FaSSGF + 20% ethanol); multitablets, MT < 7% (FaSSGF)	Compression	[7]
Eudragit L100-55	90–140	Polymeric matrix	**	*	Compression	[96]
Eudragit RS PO	80–125					

* release studies were not performed; ** do not use drug. ATBC, acetyl tributyl citrate; FaSSGF, fasted state simulated gastric fluid; K12, Plasdone K-12 povidone; S630, Plasdone S-630 copovidone; TEC, triethyl citrate; ^$^ detailed release data are shown due to the lack of information in some original studies impairing the classification of the drug release behavior (immediate, controlled, or delayed).

**Table 4:** Other formulations produced by HME using Eudragit polymers.

Eudragit Type	Extrusion Temperature (°C)	Polymer Role	Drug	Pharmaceutical Form	Release Data ^$^	Reference
Eudragit E PO	25–125	Polymeric matrix	Furosemide and naproxen	Polyelectrolyte complexes	18% in 2 h, water; 100% in 30′, if NaCl 0.15M is added at the start of release study; No release up to 1 h and 100% in 2 h, if NaCl 0.15 M is added after 1 h; >60% in 2 h, if NaCl 0.002 M is added at the start; and <10% in 30′, 20% in 1 h and 100% in 2 h if NaCl 0.002 M is added after 30′ and NaCl 0.15 M after 1 h	[97]
	70–110	Polymeric matrix/Taste-masker agent	Efavirenz	Pellet	90% in 30′ (10%, 25% and 50% of drug); <70% in 60′ (60% and 70% of drug)	[98]
	110	Polymeric matrix	Rifampicin	Pellet	100% in 10′	[99]
	90–130	Polymeric matrix	Ibuprofen	Extrudates	≅70% in 1 h in pH 1.2 and 100% in 2 h in pH 6.8 (30% and 50% drug); ≅20% in 1 h in pH 1.2 and < 60% in 2 h in pH 6.8 (70% drug)	[100]
	92	Polymeric matrix	Ibuprofen	Cocrystal suspension	11.64% in 3 h	[101]
	130	Polymeric matrix/Taste-masker agent	Caffeine citrate	Extrudate	<3.5% in 30” in artificial saliva; ≅99% in 12 h in water	[102]
	105–120	Polymeric matrix	Indomethacin	nd	*	[103]
	120–140	Polymeric matrix	Nimodipine	Pellet	100% in 30′ (90% EPO; EPO:HPMC 2:1 and 2:3); 85% in 30′, (EPO:HPMC, 1:1)	[104]
	120	Polymeric matrix	Ibuprofen	Dry suspension	90% in 5′	[105]
	135–145	Polymeric matrix/Taste-masker agent	Tilmicosin	Extrudates	<2% in 30″ in artificial saliva; >80% in 30′ in 0.1 M HCl	[106]
Eudragit E100	140	Polymeric matrix	Nimodipine	Pellet	85% in 30′	[107]
Eudragit E100 PO	85–130	Polymeric matrix	Ketoprofen	Extrudates	100% in 30′ (drug:polymer, 10:90, 30:70, 50:50); ≅80% in 2 h (drug:polymer:PVP, 30:50:20); ≅60% in 2 h (drug:polymer:PVPVA, 30:50:20); >80% in 2 h (drug:polymer:HPMC 30:50:20);	[108]
Eudragit FS 100	90	Polymeric matrix	Moxifloxacin hydrochloride	Ocular insert	>70% in 24 h	[109]
Eudragit L100	35–78	Release modifier	Acetaminophen, ibuprofen, phenazon and tramadol-HCl	Pellet	100% in 1 h	[110]
Eudragit L100-55	100–125	Polymeric matrix	Esomeprazole and naproxen	Fixed-dose combination extrudate	No drug release in 2 h in 0.1 N HCl, 100% in 12 h in pH 6.8	[111]
Eudragit RL PO	120–160	Polymeric matrix	Domperidone	Film	49% in 2 h	[112]
	120–135	Polymeric matrix	Noscapine	Sustained release extrudate	10.93% in 2 h in pH 1.2 and 22.25% in 24 h in pH 6.8 (formulation without CA); 13.68% in 2 h in pH 1.2 and 70.99% in 24 h in pH 6.8 (with 10% CA);	[35]
Eudragit RS	20–160	Polymeric matrix	Theophylline	Orodispersible film	80% in > 120′ (particle size < 315 μm); ≅85% in 1000′ (500–715 μm); ≅50% in 1000′ (>1000 μm)	[113]
Eudragit RS PO	70–140	Polymeric matrix	Metropolol tartrate	Extrudates	*	[114]
	90–120	Polymeric matrix	Venlafaxine HCl	Extrudates	*	[115]
	45–150	Polymeric matrix	Venlafaxine HCl	Extrudates	72% to 95% in 8 h	[116]
	40–128	Polymeric matrix	Metropolol succinate	Floating multiparticulates	100% in 12 h	[117]
	90–100	Polymeric matrix	Ibuprofen	Trandermal film	RS (100%) 21.6% in 24 h; RS:Suc (60:10) 21.8% in 24 h; RS:MC (60:10) 25.5% in 24 h; RS:MC (10:60) 99% in 24 h; RS:XG (60:10) 82.7% in 24 h; RS:XG (10:60) 94.4% in 24 h; RS: Pol (60:10) 42.9% in 24 h; RS:Gel (60:10) 58.2% in 24 h; RS:Gel (50:20) 98.1% in 4 h;	[11]
	90–120	Polymeric matrix	Velafaxine	Pellet	≅35% in 2 h (citric acid 10%); ≅50% in 2 h (citric acid 20%); ≅70% in 2 h (Lutrol 10%); ≅90% in 2 h (Lutrol 20%);	[118]
Eudragit S100	120	Polymeric matrix	5-Aminolevulinic acid hexyl-ester	Extrudates	<5% in 2 h in 0.1 M HCl, 21% in 6 h in pH 7.4	[119]
			Methylene blue		No drug release in 2 h in 0.1 M HCl, 31% in 6 h in pH 7.4	
			Meso-tetra porphine tetra tosylate		No drug release in 2 h in 0.1 M HCl, 50% in 6 h in pH 7.4	
	100–145	Polymeric matrix	Ibuprofen	Pellet	2.5% EC ≤ 18% in 6 h; 100% in 12 h (pellet 3 mm); 5% EC ≤ 18% in 6 h; 100% in 24 h (3 mm)	[9]
			Ketoprofen		2.5% EC ≤ 20% in 6 h (1, 2 and 3 mm); 100% in 12 h (1 mm); 100% in 14 h (2 mm); 100% in 16 h (3 mm); 5% EC ≤ 20% in 6 h; 100% in 14 h (1 mm); 100% in 16 h (2 mm); 100% in 22 h (3 mm);	
Eudragit E PO/RS PO	120–140	Polymeric matrix	Metformin	Floating tubes	Sustained **	[120]
Eudragit L100		Polymeric matrix	Metropolol succinate	Extended release delivery system	<50% in 20 h	[48]
Eudragit S100						
Eudragit L100/L100-55		Polymeric matrix/Release modifier			<3% in 2 h in 0.1 N HCl, 100% in 24 h in pH 6.8	
Eudragit S100/L100-55 (28.2% + 23.1%)					<3% in 2 h in 0.1 N HCl, ≅30% in 24 h in pH 6.8	
Eudragit S100/L100-55 (23.1% + 28.2%)					<3% in 2 h in 0.1 N HCl, ≅70% in 24 h in pH 6.8	
Eudragit S100/L100-55 (25.6% + 25.6%)					<3% in 2 h in 0.1 N HCl, ≅80% in 24 h in pH 6.8	
Eudragit L100/P303					<40% in 2 h in 0.1 N HCl, 100% in 11 h in pH 6.8	
Eudragit S100/P303					<40% in 2 h in 0.1 N HCl, 100% in 15 h in pH 6.8	
Eudragit RL PO	10–110	Polymeric matrix	Theophylline	Floating pellet	*	[121]
Eudragit RS PO					Ranging between 24% to 96.2% in 18 h ***	
Eudragit RL PO	140–150	Polymeric matrix	Carbamazepine and theophylline	Extrudates	≅85% in 8 h (10% Theo); ≅100% in 1 h (30% Theo); >90% in 12 h (10% CB); ≅90% in 8 h (30% CB)	[38]
Eudragit RS PO					≅20% in 24 h (10% Theo); ≅70% in 12 h (30% Theo); ≅40% in 12 h (10% CB); ≅70% in 12 h (30% CB)	
Eudragit RL PO/RS PO (30:60)					≅20% in 24 h (10% Theo); ≅80% in 12 h (10% CB)	
Eudragit RL PO/RS PO (45:45)					≅20% in 24 h (10% Theo); > 90% in 12 h (10% CB)	
Eudragit RL PO/RS PO (60:30)					≅50% in 12 h (10% Theo); >90% in 12 h (10% CB)	
Eudragit RL PO/RS PO (24:46)					>90% in 8 h (30% Theo); ≅75% in 12 h (30% CB)	
Eudragit RL PO/RS PO (35:35)					>90% in 4 h (30% Theo); ≅80% in 12 h (30% CB)	
Eudragit RL PO/RS PO (46:24)					>90% in 4 h (30% Theo); ≅85% in 12 h (30% CB)	

* release studies were not performed; ** in this study, ten different formulations were produced, with different EE and ERS ratio; release studies in 3 different pH media were performed, in all of them, sustained release were observed; *** in this study 11 different formulations were produced, but the exact content of ERS could not be identified; ^$^ detailed release data are shown due to the lack of information in some original studies impairing the classification of the drug release behavior (immediate, controlled, or delayed); nd, not clearly identified. CA, citric acid; CB, carbamazepine; EC, ethyl cellulose; Gel, gelucire 44/14; HPMC; hydroxypropyl methylcellulose; MC, methyl cellulose; NaCl, Sodium chloride; P303, Polyox™ WSR 303; Pol, poloxamer; PVP, polyvinylpyrrolidone; PVPVA, poly(vinylpyrrolidone-co-vinyl acetate); Suc, sucrose; Theo, theophylline; XG, xanthan gum.

**Table 5:** 3D printed products by FDM technique using Eudragit polymers.

Eudragit Type	Nozzle Temperature (°C)	Polymer Role	Drug	Pharmaceutical Form	Release Data ^$^	Reference
Eudragit E PO	150	Polymeric matrix	Felodipine	Disc	84.3% in 30′ (HCl pH 1.2); 100% in 6 h (PBS pH 6.8)	[130]
	135	Polymeric matrix	5-ASA, theophylline, captopril and prednisolone	Tablet	85% in 30′	[131]
	135	Polymeric matrix	Sodium warfarin	Tablet	>80% in 45′	[132]
	230	Polymeric matrix	Acetaminophen	*	**	[133]
	135	Polymeric matrix	Hydrochlorothiazide	Tablet	100% in 60′	[134]
	135	Polymeric matrix	Enalapril maleate and hydrochlorothiazide	Tablet	100% in 60′	[135]
	160–175	Polymeric matrix	Pramipexole	Tablet	>90% in 60′ (EPO:Poliox N10); >90% in 90′ (EPO:Poliox N80, 50:50); >90% in 60′ (EPO:Poliox N80, 60:40); >90% in 25′ (EPO:Poliox N80, 70:30);	[136]
	135–200	Polymeric matrix	Carvedilol	Tablet	80% in 11 h (Aff 15: EPO, 60:15)	[137]
	200	Taste-masking agent	Caffeine citrate	Donut shaped tablet	>80% in 60′ (10% infill); ≅75% in 120′ (50% infill); ≅50% in 120′ (100% infill)	[1]
	160–165	Polymeric matrix	Lumefantrine	Tablet	90% in 30′ (65% infill); 78% in 30′ (80% infill); 69% in 30′ (100% infill)	[138]
	200	Polymeric matrix	Theophylline	Tablet	85% in 50′ (10% drug); 85% in 30′ (30% drug); 85% in 48′ (60% drug)	[139]
Eudragit FS30D	***	Delaying release polymer	Theophylline	Printfill	2.3% in 2 h (pH 1.2); 80% in 8 h (pH 7.5)	[140]
Eudragit L100	190	Coating	Budesonide	Tablet	<5% in 2 h (0.1 N HCl); ≅45% in 5 h30′ (pH 5.6–7.4); ≅85% in 10 h (pH 6.5)	[141]
	200	Polymeric matrix	Acetaminophen	Tablet	<10% in 24 h	[142]
Eudragit L100-55	185	Enteric polymer	Theophylline, budesonide and diclofenac	Tablet	65% in 2 h in pH 1.2 and ≅100% in 150′ in pH 6.8 (0.17 mm shell); 75% in 2 h in pH 1.2 and ≅100% in 150′ in pH 6.8 (0.35 mm); <3% in 2 h in pH 1.2 and ≅100% in 360′ in pH 6.8 (0.52, 0.7 and 0.87 mm)	[143]
	167, 172 and 175	Enteric polymer	Riboflavine-5′-phosphate	Capsule	5% in 2 h in pH 1.2 and 87% in 45′ in pH 6.8 (layer height 100 μm); 5% in 2 h in pH 1.2 and 100% in 45′ in pH 6.8 (200 μm); 23% in 2 h in pH 1.2 and 100% in 45′ in pH 6.8 (300 μm)	[144]
	178	Polymeric matrix	Acetylsalicylic acid and simvastatin	Polypill	0% (pH 1.2); 100% in 45′ (pH 6.8)	[145]
Eudragit RL PO	170	Polymeric matrix	Metformin	Tablet	100% in 8 h (single screw filament); 91.76% in 9 h (twin screw filament)	[146]
	180	Polymeric matrix	Theophylline	Tablet	85.93% in 2 h (ERL-PEG 10%); 10.66% in 2 h (ERL-SA 7%)	[147]
	180	Polymeric matrix	Theophylline	Tablet	>90% in 24 h (10%, 15%, 20%, 25% and 30% infill); 30% in 11 h (75% infill)	[148]
	195	Polymeric matrix	Theophylline	Caplet	100% in 10 h (HPC:ERL:PEG:drug, 4:4:1:1); 100% in 4 h (HPC:ERL:PEG:drug, 5:3:1:1 and 6:2:1:1)	[34]
	205–215	Polymeric matrix	Indomethacin	*	**	[149]
Eudragit RL 100	170	Polymeric matrix	Deflazacort	Tablet	≅50% in 24 h (without mannitol); ≅70% in 24 h (mannitol); >80% in 24 h (mannitol and 50% infill)	[150]
Eudragit RS PO	155	Polymeric matrix	Quinine	Implant	3.7% in 78 days	[151]
	180	Polymeric matrix	Carvedilol	Floating tablet	Basket—≅90% in 24 h (C1); ≅65% in 24 h (C2); ≅60% in 24 h (C3 and C4); Paddle—≅95% in 24 h (C1); ≅65% in 24 h (C2); ≅70% in 24 h (C3); ≅60% in 24 h (C4); Tapped basket— ≅100% in 24 h (C1, C2, C3 and C4)	[152]
	200	Polymeric matrix	Quercetin	Patch	≅9% in 70 h (drug:PVP40:ERS:TEC, 1:49:38:12); ≅7% in 24% (1:37:50:12); ≅12% in 24 h (1:25:62:12)	[153]
	170	Polymeric matrix	Octreotide	Capsule	No release in acid medium (0.1 M HCl); >80% in 30′ (PBS pH 6.8)	[154]
Eudragit RS 100	160–180	Polymeric matrix	Acetaminophen	Capsule	85% in 8′	[155]
Eudragit E	140	Polymeric matrix	Theophylline	Tablet	>90% above 40′	[156]
Eudragit RL 100	170				≅80% in 18 h	
Eudragit RS 100	150				≅10% in 18 h	
Eudragit RL 100/RS 100	150				≅50% in 18 h	
Eudragit E PO	175	Polymeric matrix	Isoniazid	Tablet	80% in 1000 min (EPO + HPC)	[157]
Eudragit L100	170				80% in 334′ (EL100 + HPC)	
Eudragit RL PO/RS PO	165				100% in 200′ (ERS + ERL + PEO + TEC)	
Eudragit E PO	135	Polymeric matrix	Theophylline and dipyridamole	Capsule	>85% in 30′	[158]
Eudragit RL	170		Theophylline		≅50% in 2 h in pH 1.2 and ≅95% in 16 h in pH 6.8 (1.6 mm shell); ≅20% in 2 h in pH 1.2 and ≅ 75% in 16 h in pH 6.8 (2 mm); ≅10% in 2 h in pH 1.2 and ≅59% in 16 h in pH 6.8 (2.4 mm)	
Eudragit RL PO	178	Release modifier	Ibuprofen	Tablet	≅10% in 24 h	[159]
Eudragit RS PO					≅7% in 24 h (20% ERS); ≅14% in 24 h (10% ERS)	
Eudragit L100-55/RL PO	160	Polymeric matrix	Furosemide	Disc	**	[160]
Eudragit L100-55/S100	182	Polymeric matrix	5-Fluoracil	Tablet	L100-55:S100, 50:25 and 0:65—No release; 78:0—50% in 180′; 73:5—50% in 270′; 68:10—no release in SF pH 1.2 and SIF pH 6.5; In pH 7.4—40% in 120′ for both coated and non-coated; 100% (non-coated) and 80% (coated) in 9 h	[161]
Eudragit RL PO/RS PO	200	Coating	Allopurinol	Expandable gastroretentive devices	≅100% in 300′	[162]

5-ASA, 5-aminosalicylic acid; Aff 15, Affinisol HME 15LV—hydroxypropyl methylcellulose; CTAB, cetyltrimethylammonium bromide; HCl, hydrochloric acid; HPC, hydroxypropyl cellulose; PBS, phosphate buffer solution; PEG, polyethylene glycol; Poliox N10, poly-ethylene oxide—PolyoxTM WSR N10; Poliox N80, poly-ethylene oxide—PolyoxTM WSR N80; SA, stearic acid; TEC, triethyl citrate. For [125]—C1—0.6 mm shell thickness and 0.3 mm layer height; C2—0.6 mm shell thickness and 0.1 mm layer height; C3—0.9 mm shell thickness and 0.3 mm layer height; C4—0.9 mm shell thickness and 0.1 mm layer height.* do not print a specific pharmaceutical form; ** release studies were not performed; *** polymer used as delaying release polymer, deposited by an injection volume filler system above the printed form; ^$^ detailed release data are shown due to the lack of information in some original studies impairing the classification of the drug release behavior (immediate, controlled, or delayed).

**Table 6:** 3D printed products by techniques other than FDM using Eudragit polymers.

Eudragit Type	Printing Tecnhique	°C Nozzle	Polymer Role	Drug	Pharmaceutical Form	Release Data ^$^	Reference
Eudragit E PO	Direct extrusion	180	Polymeric matrix	Dutasteride	Tablet	≅100% in 40′ (tube); ≅100% in 120′ (pyramid); ≅80% in 120’ (cube); ≅70% in 120‘ (hemisphere)	[163]
Eudragit RL PO	Direct extrusion	90	Polymeric matrix	Ketoprofen and nicotine	Patch	80% in 4 h (KP); 80% in 30′ (NT)	[164]
Eudragit RS PO						20% in 4 h (KP); 60%, in 1 h (NT);	
Eudragit RL PO/RS PO						30% in 4 h (KP:ERL:ERS); 95%, in 1 h (NT:ERL:ERS)	
Eudragit RL100	Direct extrusion	90	Polymeric matrix	Theophylline	Tablet	≅30% in 2 h in pH 1.2 and 80% in 12 h in pH 6.8	[165]
Eudragit RS100		80				≅5% in 2 h in pH 1.2 and 25% in 12 h in pH 6.8	
Eudragit RL100/RS100		95–110				≅25% in 2 h in pH 1.2 and 60% in 12 h in pH 6.8 (ERL:ERS, 75:25); ≅22% in 2 h in pH 1.2 and 55% in 12 h in pH 6.8 (ERL:ERS, 50:50); ≅15% in 2 h in pH 1.2 and 45% in 12 h in pH 6.8 (ERL:ERS, 25:75)	
Eudragit L100-55	Selective laser sintering	-	Polymeric matrix	Acetaminophen	Printlet	18% in 2 h; ≅60% in 6 h; ≅90% in 12 h (5% drug); 14% in 2 h; ≅60% in 6 h; ≅100% in 12 h (20% drug); 6% in 2 h; ≅60% in 6 h; ≅100% in 12 h (35% drug);	[166]
Eudragit L100-55	Selective laser sintering	-	Polymeric matrix	Acetaminophen	Tablet	17% in 2 h in HCl 0.1 M; and 100% in 12 h in pH 5.5 (cylindrical); 70% in 2 h (gyroid)	[167]
Eudragit RL						95% in 24 h (cylindrical); 100% in 2 h (gyroid)	

KP, ketoprofen; NT, nicotine; ^$^ detailed release data are shown due to the lack of information in some original studies impairing the classification of the drug release behavior (immediate, controlled, or delayed).
